# Raman Spectroscopic Analysis Reveals Abnormal Fatty Acid Composition in Tumor Micro- and Macroenvironments in Human Breast and Rat Mammary Cancer

**DOI:** 10.1038/srep32922

**Published:** 2016-09-06

**Authors:** Sixian You, Haohua Tu, Youbo Zhao, Yuan Liu, Eric J. Chaney, Marina Marjanovic, Stephen A. Boppart

**Affiliations:** 1 Beckman Institute for Advanced Science and Technology, University of Illinois at Urbana-Champaign, Illinois, USA; 2 Department of Bioengineering, University of Illinois at Urbana-Champaign, Illinois, USA; 3 Department of Electrical and Computer Engineering, University of Illinois at Urbana-Champaign, Illinois, USA; 4 Department of Internal Medicine, University of Illinois at Urbana-Champaign, Illinois, USA.

## Abstract

Fatty acids play essential roles in the growth and metastasis of cancer cells. To facilitate their avid growth and proliferation, cancer cells not only alter the fatty acid synthesis and metabolism intracellularly and extracellularly, but also in the macroenvironment via direct or indirect pathways. We report here, using Raman micro-spectroscopy, that an increase in the production of polyunsaturated fatty acids (PUFAs) was identified in both cancerous and normal appearing breast tissue obtained from breast cancer patients and tumor-bearing rats. By minimizing confounding effects from mixed chemicals and optimizing the signal-to-noise ratio of Raman spectra, we observed a large-scale transition from monounsaturated fatty acids to PUFAs in the tumor while only a small subset of fatty acids transitioned to PUFAs in the tumor micro- and macroenvironment. These data have important implications for further clarifying the macroenvironmental effect of cancer progression and provide new potential approaches for characterizing the tumor micro- and macroenvironment of breast cancer in both pre-clinical animal studies and clinical applications.

Over the last century, studies of epithelial cancers have been mainly focused on the alterations of the epithelium and the tumor microenvironment (surrounding tissue under direct effect of tumor) and have provided invaluable insights on the development, diagnosis, and treatment of cancer^1^,^2^,^3^. In recent years, scientists and clinicians have increasingly begun to expand their investigations from the local tumor microenvironment to a more global tissue macroenvironment (tissue at a large distance from the primary tumor), appreciating the active participation of a complex array of host factors in tumor progression^4^,^5^. It has been shown that the macroenvironmental regulation of cancer occurs at the genetic, proteomic, and metabolic levels to elicit carcinogenesis, metastasis, and progression^4^,^6^,^7^. Understanding how tumors interact with the macroenvironment is likely to have a major impact on our understanding of the complex mechanisms underlying cancer development, as well as establish links between cancer and metabolic diseases such as obesity and diabetes.

Altered lipid metabolism is a hallmark of breast cancer^8^,^9^,^10^. An increase in polyunsaturated fatty acids (PUFAs) has been positively associated with the aggressiveness of cancer cell lines^11^ and the promotion of tumorigenesis^12^. It has been shown that higher PUFA levels increases the risk of metastasis in cancer patients by increasing estrogen levels^12^,^13^, expression of cancer-promoting genes such as PAI-1^14^,^15^, and the adherence of circulating tumor cells to blood vessel walls and remote organs^16^. Although it is known that lipid metabolism is drastically altered in cancer cells, it is not known whether such effects occur in more distant tissue sites, i.e. the macroenvironment. Evidence from other lipid-related studies strongly suggests that metabolic diseases such as obesity and diabetes can interfere with several lipogenic regulatory pathways, which in turn may result in an increased predisposition for cancer^17^,^18^. Despite the importance of these studies to the development of new interventions and therapeutics, further investigations are hindered by the lack of reliable biochemical signatures for the tumor macroenvironment.

Here, we report that by using Raman spectroscopy, we are able to characterize both the tumor micro- and macroenvironments in human breast and rat mammary cancer. Raman spectroscopy is a noninvasive, label-free, chemical-specific technique^19^,^20^. It is widely used for the diagnosis of various cancers by analyzing the abundance of chemical species within the tumor^21^,^22^,^23^; however, its ability to characterize chemical changes outside the tumor, such as tumor micro- and macroenvironment, has been underappreciated. As Raman signals from one single point arise from all chemical components present in the probed volume, a considerable part of the rich information carried within the signal can be easily compromised by the complex mixtures of chemicals, as well as by the noise and background from the instrument and sample. Thus, we propose that if these effects are minimized, there is a chance that we could identify previously unseen changes in the tumor micro- and macroenvironment.

The objective of this study is to characterize the alteration of PUFA abundance in the tumor micro- and macroenvironment in human breast and rat mammary cancer by utilizing the power of Raman micro-spectroscopy. Raman micro-spectroscopy was chosen in this study to avoid the volume averaging effect and to ensure optimal spatial resolution and chemical specificity^24^. After data acquisition, a pattern recognition algorithm was employed to match the Raman spectra to a predefined library of fatty acids so that interference from unwanted chemical species would be minimized. In the pre-clinical study, increased PUFA levels were identified at the tumor sites, and within the tumor micro- and macroenvironment of the mammary gland in cancerous rats, compared with mammary tissue from healthy rats. This observation was further validated in human subjects diagnosed with invasive ductal carcinoma compared with cancer-free subjects, which may provide new insights into the mechanisms of how breast cancer regulates its environment, as well as identify potential approaches for the assessment of the micro- and macroenvironment in both human subjects and pre-clinical rat models.

## Results

### Increased PUFA Levels in the Tumor, Tumor Micro- and Macroenvironment of Tumor-Bearing Rats

**Figure 1:**
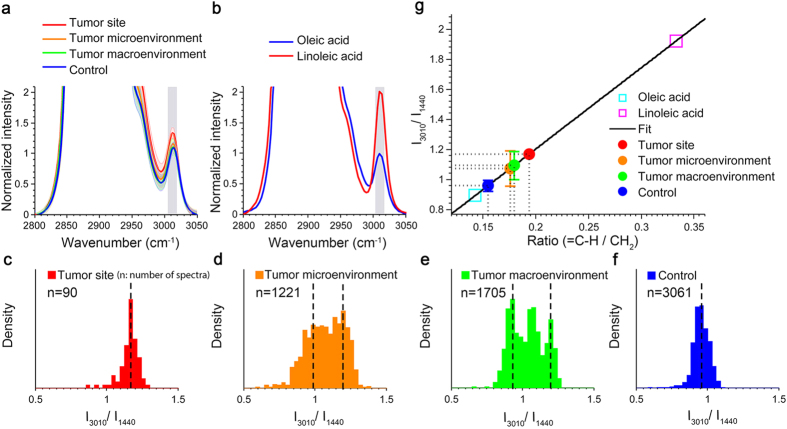
PUFA levels increase in the tumor site, tumor microenvironment and tumor macroenvironment of tumor-bearing rats, compared with healthy mammary tissue from control group. (**a)** Comparison of average Raman spectra of fatty acids in the mammary tissue from tumor-bearing rats, and healthy mammary tissue from control rats. Shaded curves show the corresponding standard deviations of all spectra acquired from each group. The major peak used for quantifying the degree of unsaturation is marked with shaded vertical bands (3010 cm^−1^). (**b)** Raman spectra of two pure reference fatty acids (oleic acid and linoleic acid) in the CH region from 2800 cm^−1^ to 3050 cm^−1^. (**c**–**f)** Density distributions of the level of unsaturation of fatty acids from four different groups (5 rats in each group): tumor, tumor micro- and macroenvironments, and control (no treatment group). (**g)** Quantitative analysis of 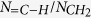 of fatty acids in each tissue sample. TheRaman measurements of the extracted fatty acids from tissue (*I*_3010_/*I*_1440_) were fitted on the linear model to predict the molecular structure (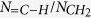). Error bars indicate the measurement standard deviations.

To examine the spatial distribution of PUFA abundance in mammary cancer, we treated one group of rats with N-Nitroso-N-methylurea (NMU) and compared the fatty acid composition of tissues at varying distances from the tumors with that of healthy mammary tissue from the other group of rats of no treatment (Supplementary Fig. S1). Using a pattern recognition algorithm (see Methods), we were able to retrieve spectra that were dominated by fatty acids (Supplementary Fig. S2). Figure 1 provides the spectral analysis of the fatty acids in the tumor sites (solid tumor region), the tumor microenvironment (mammary tissues that were immediately proximal to tumor and within 1 cm away from the center of a solid tumor), the tumor macroenvironment (mammary tissues that were more than 3 cm away from the center of a solid tumor), and control samples (healthy mammary tissue from a cancer-free rat). Consistent with previous results, Raman spectra of fatty acids in normal mammary tissue were dominated by Raman features of oleic acid, a monounsaturated fatty acid that is the most abundant fatty acid in mammalian cells^25^ (Fig. 1a,b). Conversely, the higher average degree of unsaturation (3010 cm^−1^) appeared to be found in the tissue of tumor-bearing rats. However, as the peak at 3010 cm^−1^ is overlapped with the tail of its strong neighbor CH band, more delicate quantification involving peak decomposition (see Methods) is required to confirm this observation.

For a better understanding of the trends observed in these spectra, quantification of the entire tissue sample was performed to analyze fatty acid composition. By Beer’s law, the ratio of the Raman intensity at 3010 cm^−1^ (=*C*−*H* stretching vibration^26^) to the intensity at 1440 cm^−1^ (CH_2_ deformation^20^), *I*_3010_/*I*_1440_, depends linearly on the ratio of the number of =*C*−*H* groups to the number of *CH*_2_ groups^27^. After peak decomposition, *I*_3010_/*I*_1440_ was measured from each spectrum to obtain histograms of the degrees of unsaturation of fatty acids in the four groups (Fig. 1c–f). We found that both tumor and control groups had a unimodal Gaussian-like distribution for *I*_3010_/*I*_1440_ values (Fig. 1c,f), with distinct mean values, suggesting a large-scale compositional shift from oleic acids (C18:1) to PUFAs or palmitoleic acids (C16:1) in solid tumors. In contrast, we observed a bimodal/trimodal distribution in the tumor micro- and macro-environments (Fig. 1d,e). The bimodal/trimodal distribution reflected the presence of two or three distinct types of fatty acid mixtures that were well-separated in space, with one mode similar to that of the control group (mostly oleic fatty acids) and the other modes similar to that of the tumor group (larger proportion of PUFAs or palmitoleic acids). These results suggest that a tumor-supportive environment and healthy tissue may differ by only a relatively small subset of fatty acids.

For a more intuitive interpretation of *I*_3010_/*I*_1440_, a linear model for predicting the degree of unsaturation was used based on the measured spectra and known chemical structure from reference fatty acids: oleic acid (C18:1) and linoleic acid (C 18:2)^27^,^28^ (Fig. 1g). The average value of *I*_3010_/*I*_1440_ was used to predict the average 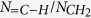 for each tissue sample. It was found that tissue samples from tumor-bearing rats exhibited a significantly (P = 3.8 × 10^−5^) higher ratio of 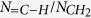 than those from the control group (mean ± standard error: 0.1548 ± 0.0035), regardless of whether they were from the solid tumor site (0.1937, no standard deviation was associated with this measurement since only one sample was qualified for fatty acid analysis as explained below), tumor microenvironment (0.1760 ± 0.0107), or tumor macroenvironment (0.1798 ± 0.0088). It is worth noting that, compared with other samples, only one solid tumor sample had a sufficiently high concentration of fatty acids for analysis because the Raman signals from the solid tumor areas tended to have greater contributions from the cell cytoplasm, cell nucleus, and collagen, but much less from fatty acids, which is consistent with what was reported in previous studies^21^,^29^.

As shown by the chemical structures in Supplementary Fig. S3, a ratio value (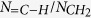) higher than 1/7 (oleic acid) indicates either an increase in the number of double bonds, which corresponds to an increase in PUFA levels, or a decrease in the number of carbons of the monounsaturated fatty acids, which implies an increased level of palmitoleic acid (C16:1)^30^. As predicted by the chemical formula and the linear model (Fig. 1g), palmitoleic acid has a ratio of 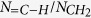
at 1/6 and *I*_3010_/*I*_1440_ at 1.125. By comparing this value to the ratio value obtained from rat tissue (Fig. 1c–f), it was observed that the value of palmitoleic acid is very close to the mean value obtained from the tumor sites, as well as the mean value of the abnormal modes in the bimodal/trimodal distribution obtained from the tumor microenvironment and the tumor macroenvironment. This comparison indicates an almost full-scale transition from oleic acid to palmitoleic acid in tumor sites if the increased abundance in palmitoleic acid was indeed the main reason for the observed Raman shifts. However, considering the fact that palmitoleic acid is only a minor component in adipose tissue in animals and humans with or without cancer present^31^, the theory of a complete transition to palmitoleic acid in cancerous tissue is implausible. The same rationale holds for tissue samples from the tumor micro- and macroenvironments. Therefore, we conclude that the significant increase in the degree of unsaturation for the fatty acids in the tumor and the tumor micro- and macro-environments is attributed to an increase in the PUFA levels.

### Increased PUFA Levels in Cancerous and Normal-Appearing Human Breast Tissue

**Figure 2:**
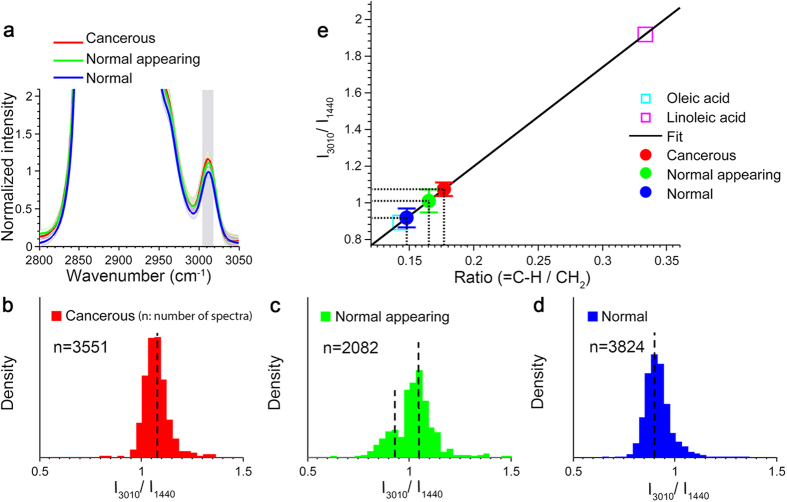
PUFA levels increase in cancerous human breast tissue, as well as normal-appearing breast tissue from subjects diagnosed with invasive ductal carcinoma, compared with breast tissue from cancer-free subjects. (**a)** Comparison of average Raman spectra of fatty acids from cancerous and normal-appearing tissue of cancer subjects, and from normal tissue of cancer-free subjects. (**b**–**d)** Density distributions of the degree of unsaturation of fatty acids from three groups of tissue samples: cancerous, normal-appearing, normal. **(e)** Quantitative analysis of 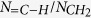 of fatty acids in each tissue sample.

Given that NMU has been shown to induce stromal alteration to promote carcinogenesis^32^, it is intuitive to question whether the macroenvironmental changes observed in the pre-clinical study were also induced by NMU instead of breast cancer development. To address this concern and also to explore the clinical potential of our finding, a similar spectral analysis was performed on human breast tissue. Figure 2a showed that the average intensities of the unsaturation band (3010 cm^−1^) of the fatty acids in the tissue samples from the cancer group appeared to be stronger than those in the healthy group, indicating possibly increased PUFA levels. Using more rigorous peak analysis (see Methods), we found that, consistent with the pre-clinical rat study, both cancerous and healthy tissue samples had a unimodal distribution of the degree of unsaturation with different mean values, suggesting a large-scale transition from monounsaturated fatty acids to PUFAs at the tumor sites and nearby tumor boundaries (Fig. 2b,d). In contrast, the group of normal appearing tissue exhibited a bimodal pattern in the distribution of the degree of unsaturation of fatty acids (Fig. 2c), which suggests the alteration of a relatively small subset of fatty acids in the tumor macroenvironment of human breast cancer.

The next step was to see if significantly increased PUFA levels accompany human breast cancer. As shown in Fig. 2e, the average value of 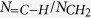 of fatty acids from healthy breast tissue was closest to that of oleic acid (0.1478 ± 0.0048 vs. 0.1429), which further confirms the predominance of monounsaturated fatty acids in healthy human breast tissue^33^. Consistent with the rat study, cancerous tissue showed a higher value of 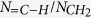 compared with normal tissue from cancer-free subjects (0.1768 ± 0.0035 and 0.1478 ± 0.0048, respectively). This observation is in line with previous experimental studies reporting that PUFAs have tumor- and metastasis-promoting effects in cultured cells, animals and human^12^,^14^,^16^. It was also observed that the normal appearing tissues from cancer subjects have significantly (*P* = 1.4 × 10^−5^) higher PUFA levels (0.1650 ± 0.0058) compared with the normal tissue from cancer-free subjects, suggesting that the regulation and alteration of lipid metabolism by cancer cells affect not only the primary tumor site and its local microenvironment, but also areas in the macroenvironment even a few centimeters away from the primary tumor.

### No Observable Correlation to the Density of Cancer Cells

**Figure 3:**
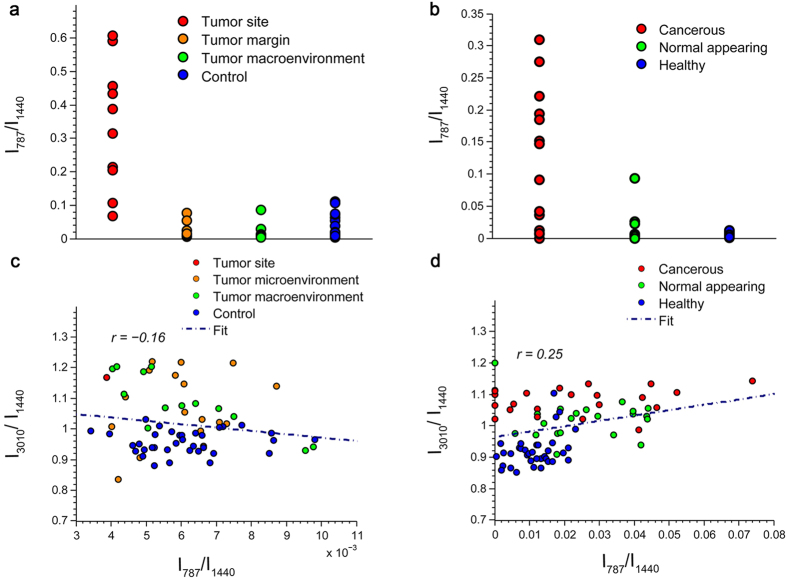
The lack of a strong DNA signal and poor correlation between DNA and PUFA levels in the tumor micro- and macroenvironment show that increased PUFA levels are not the consequence of disseminated cancer cells in both the rat and human tissue. One mean DNA concentration value was generated for each specimen and grouped in terms of their location and cancer status. (**a)** Scatterplot of relative DNA concentration of different tissue sites in rats and in (**b)** human. (**c)** Correlation plot of relative DNA concentration and 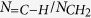 of fatty acids in rats and in (**d)** human.

Since it has been suggested that increased PUFA levels can be positively associated with the presence of metastatic cancer cells^11^, we considered the possibility that the observed alteration of fatty acid composition in the tumor macroenvironment could be a local influence resulting from a small number of disseminated cancer cells, instead of a macroenvironmental effect of cancer progression. To address this, evaluation of the correlation between PUFA levels and the abundance of cancer cells is needed. First, abundance of cancer cells at different tissue sites was measured. The 787 cm^−1^ Raman peak (DNA: O-P-O, cytosine, uracil, thymine^20^), a signature for DNA, cell nuclei, and cancerous tissue, was used to quantify the relative concentration of nucleic acids present in the tissue^34^. High concentrations of DNA were only observed at the tumor sites (Fig. 3a,b), which is consistent with many reports of using Raman spectroscopy for tumor detection^11^,^34^. Secondly, the effect of the density of cancer cells on the concentration of PUFAs in tissue was tested. The poor correlation between the relative DNA concentration and PUFA accumulation (Fig. 3c,d), together with the absence of strong DNA bands in the tumor micro- and macroenvironment, demonstrates that the observation of increased production of PUFAs is independent from the presence of densely-accumulated cancer cells and likely a realistic reflection of global macroenvironmental changes in lipid metabolism.

### High Correlation to Fingerprint Region of Raman Spectrum

**Figure 4:**
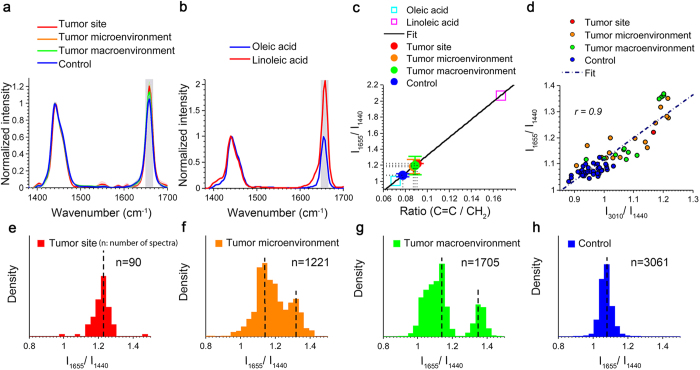
Analysis in the fingerprint region also shows increased PUFA levels in the tumor and in the micro- and macroenvironments in the rat mammary gland. (**a**) Comparison of average Raman spectra of fatty acids from tumor sites, the micro- and macro tumor environments of tumor-bearing rats, and the healthy tissue from control rats. (**b**) Raman spectra of two pure reference fatty acid (oleic acid and linoleic acid) in the fingerprint region from 1400 cm^−1^ to 1700 cm^−1^. (**c**) Quantitative analysis of 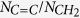 of fatty acids from different tissue sites. (**d**) Correlation plot of Raman intensity at 1665 cm^−1^ and 3010 cm^−1^ from fatty acids of various sites in cancer and control rats. (**e**–**h**) Density distributions of the degree of unsaturation of fatty acids from four different groups (5 rats in each group): tumor, micro- and macroenvironments, and control (no treatment group).

It was also investigated if our findings from the Raman CH stretching band region (2800–3100 cm^−1^) could be extended to the fingerprint vibration region (1200–1800 cm^−1^), which is a commonly used spectral range in cancer diagnosis. Figure 4 shows the analysis of 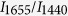, the ratio of the Raman intensity of the C=C stretching vibration to the intensity of the *CH*_2_ deformation^27^. As expected, results from the two spectral ranges in the rat study are strongly correlated and demonstrate a similar trend that the primary tumor, the tumor microenvironment, and the tumor macroenvironment have significantly (*P* = 8.2 × 10^−8^) higher PUFA levels, compared with the healthy tissue environment. In previous analyses, the Raman CH vibration was chosen over the fingerprint vibration region because β-carotene is often found in conjunction with fat throughout the human breast^35^, and produces strong Raman signals in the fingerprint vibration region but not in the CH vibration region^33^,^36^ at the excitation wavelength of 532 nm (Supplementary Fig. S2).

## Discussion

Altered lipid metabolism has been extensively investigated in cancer cells^11^,^16^, but not in their micro- or macroenvironment. This imbalance of attention is due in part to the belief that normal appearing tissue, which is usually more than 5 cm away from the tumor sites (in human), could be assumed to be similar to healthy tissue and used as a control group in comparison studies. However, our results suggest that subtle but reproducible differences exist in the fatty acid content between healthy tissue from cancer-free subjects and normal appearing tissue from subjects with cancer. By optimizing high signal-to-noise ratio (SNR) and minimizing the effect of volume-averaging of chemicals during Raman data acquisition and processing, significantly increased PUFA levels were identified in the tumor, as well as in the tumor micro- and macroenvironments in both human tissues and pre-clinical rat models. These data support the concept of micro- and macroenvironmental regulation in mammary tissues of humans and rat models with breast carcinoma^4^,^5^,^7^. Our findings are likely to provide new insights into the mechanisms of breast cancer development and also suggest potential approaches for the assessment of the macroenvironment in human tissues and rat models.

The increases of PUFA levels in the normal appearing tissue from human subjects and pre-clinical rat models are in line with reports that have shown the cancer-promoting effects of PUFAs in the macroenvironment^7^. It is of note that different PUFA families have different effects on breast cancer. Indeed, n-6 PUFAs have been shown to promote tumorigenesis via increasing circulating estrogenic compounds levels^13^, cell proliferation rate^37^, and inflammation^38^, while n-3 PUFAs have been shown to be responsible for suppressing tumor growth by decreasing circulating estrogenic compound levels^13^, breast cancer cell growth^39^, and HER2 expression^40^. These studies support our findings and suggest that the majority of the PUFA content we investigated here are n-6 PUFAs^11^. Nevertheless, further studies are needed to confirm this.

More specifically, a significantly increased abundance of n-6 linoleic acids at tumor sites has been documented in clinical studies involving patients with breast cancer^41^. However, the increased PUFA levels observed in the tumor micro- and macroenvironment are unexpected and even appear contradictory to previous studies that have suggested the variation in fatty acid content between the tumor macroenvironment and healthy tissue is negligible^42^. According to the distributions shown in Figs 1d,e and 2c, the tumor micro- and macroenvironment and healthy tissue only differ by a relatively small subset of fatty acids, compared with the large-scale transition of fatty acid content at the tumor sites. Differentiation by analytical methods used in the past may have been hindered by the volume averaging effect and relatively low SNR since speed and portability are usually prioritized in clinical studies.

To date, studies on the links between lipid metabolism and breast cancer have been largely limited to murine models, mainly because cells involved in adipogenic regulatory networks are difficult to obtain and culture for long periods of time^43^. Lipid metabolism has been shown to differ greatly between human and murine models so we must be cautious to extend our observations in murine models to humans^44^. Nevertheless, the consistency between the pre-clinical rat study and the human study suggests that the NMU-induced rat mammary tumor model may have high potential for being used as a future pre-clinical model for studies involving PUFA production in the tumor sites, the microenvironment, and the macroenvironment.

In addition to the similar general trend of increasing PUFA levels in both the rat tumor model and human breast cancer subjects, it is also interesting to observe that the rat tumor model exhibits a trimodal distribution for the tumor macroenvironment (Fig. 1e), while a bimodal distribution is observed for the normal appearing regions in the human breast cancer subjects (Fig. 2c). It is possible that this difference in the distribution pattern could be attributed to differences in lipid metabolism between the rat model and the human subjects. A larger sample size and a more in-depth experimental approach are needed in a future study to investigate this possibility.

Furthermore, our study demonstrates the capability of Raman micro-spectroscopy to identify the subtle but reproducible chemical changes in the tumor micro- and macroenvironment in human tissues and rat models with breast cancer. Considering the rapid advances in the development of portable Raman spectroscopy^23^,^45^ and coherent Raman imaging microscopy^46^,^47^, our proposed strategy may help future applications related to intraoperative detection, prognosis, and assessment of macroenvironment-targeted therapies. Moreover, as a typical Raman spectrometer offers the best performance in either the fingerprint or CH region, the high correlation demonstrated in the fatty acid analysis between these regions gives researchers the freedom to choose either vibration band of interest.

Our data suggest that breast cancer is not just a localized event centered on carcinoma cells in the primary tumor, but rather a more global process involving tissue surrounding and even centimeters away from the primary tumor. Our observation of increased PUFA levels in the macroenvironment of breast cancer, together with previous reports that have shown the positive association between PUFAs and obesity^48^, raises the intriguing possibility that increased PUFA levels in the tumor macroenvironment might be one of the biochemical links between breast cancer and obesity. Therefore, the altered lipid metabolism should not be considered exclusively as an effect of cancer progression, but also possibly as a pre-disposition for breast carcinogenesis and metastasis^15^,^40^,^48^.

A clear limitation of this study is the small number of animals and human tissue specimens involved in the statistical analysis. However, we wish to emphasize that the main purpose of this study is to propose a new hypothesis in the macroenvironmental regulation during carcinogenesis and an approach for detection. To establish the clinical significance of our findings, chemical analysis of a larger number of samples is needed. Another important future direction is to consider the heterogeneity of breast cancer and take the following factors into account in the measurement of PUFA levels: age, diet, lipid profile (low-density lipoproteins, high-density lipoproteins, and cholesterol levels), menopausal status, HER2 status, and use of hormonal methods of birth control including oral contraceptives or hormone-delivery skin patches.

In short, our study shows increased PUFA levels in the tumor, and tumor micro- and macroenvironment of human breast cancer and rat mammary cancer, which has important implications for further clarifying the macroenvironmental effect of cancer progression. Our findings also suggest that there may be high potential in using Raman scattering for characterizing the tumor micro- and macroenvironment of breast cancer in both pre-clinical animal studies and clinical applications. This could furthermore influence current therapeutic approaches for breast cancer and related metabolic diseases.

## Methods

### Animal Protocol

Animal procedures were conducted in accordance with a protocol approved by the Institutional Animal Care and Use Committee at the University of Illinois at Urbana-Champaign. To induce mammary tumors in the female Wistar-Furth rats (Harlan, IN), NMU (distilled water as the vehicle; 12.5 mg/mL; Sigma, St. Louis, MO) was injected intraperitoneally at a concentration of 55 mg/kg into the left side of the abdomen when the animals were 7 weeks old. One week later, the same amount of NMU was injected intraperitoneally into the right side of the abdomen. At approximately 12 weeks of age, when the mammary tumors became palpable, primary tumor sites and non-tumor mammary tissue at different distances from the primary tumors were removed and fresh tissue samples were immediately imaged within 12 hours after surgery (Supplementary Fig. S1). The status of each tissue sample was examined and validated by pathologists with histology after the acquisition of Raman spectra. More specific information about the number of samples can be found in Table 1.

### Human Breast Tissue

This study was conducted in accordance with a protocol approved by the Institutional Review Boards at the University of Illinois at Urbana-Champaign and Carle Foundation Hospital, Urbana, IL. All tissue samples were obtained from subjects who preoperatively signed an informed consent permitting the investigational use of their tissue. Fresh samples of human breast tissue were obtained from Carle Foundation Hospital and the National Development and Research Institutes, Inc and imaged between 12–36 hours after surgery. Tissue was stored in a cool saline solution at a temperature of 2–8 °C before imaging. Normal breast tissue samples were obtained from subjects undergoing breast reduction surgery. Cancerous breast tissue samples were obtained from subjects undergoing mastectomy and diagnosed by a board-certified pathologist as invasive ductal carcinoma and containing both primary tumor and non-tumor tissue that bordered the tumor (Supplementary Fig. S1). Normal appearing breast tissue samples from breast cancer subjects were obtained from areas that were at least 5 centimeters away from the removed primary tumor and were labeled as normal by pathologists. The distance of 5 centimeters was chosen to delineate the boundary between the normal appearing tissue and the potentially cancerous tissue based on the experience of our collaborating pathologists as well as previous work by Moinfar *et al*. in ref. 4, in which the normal-appearing tissue (referring to the tumor macro-environment in this article) was defined as tissue components “away (at least 15-mm distance) from the breast cancer tissue”. After the acquisition of Raman spectra, the tissue samples were examined and validated by a board-certified pathologist using standard hematoxylin and eosin-stained histopathology. More specific information about the human tissue samples can be found in Table 2.

### Data Acquisition

A continuous-wave diode-pumped solid-state laser (Ventus, Laser Quantum) operating at a 532 nm center wavelength was used as an excitation source in a commercial confocal Raman microscope system (Horiba LabRAM HR 3D, Horiba). The beam was directed into a 0.25 N.A. 10× objective with a working distance of 10.6 mm, resulting in a laser spot size of 2.6 μm at the focus. The beam out of the objective was focused directly onto the tissue with a power of ~20 mW. Back-scattered light was collected by the same objective and sent to a spectrometer. The collected spectral range was 500 to 3500 cm^−1^ at a spectral resolution of 3 cm^−1^. Wavenumbers were calibrated with spectroscopic features from pure oleic acid by linear fitting. A bright-field microscope was used to focus and choose regions of interest. Because SNR is crucial for identifying the subtle alterations in the tumor micro- and macroenvironment, 532 nm was chosen for excitation, as the Raman intensity is inversely proportional to the fourth power of the laser wavelength. The main disadvantage associated with using shorter wavelengths is the presence of strong autofluorescence in the Raman spectra. Nevertheless, we found that the autofluorescence from the sample can largely be quenched by 500-ms illumination with focused excitation light at each point prior to data acquisition.

Raman spectroscopy measurements were performed in a two-dimensional grid with a step size of 100 μm and a grid size that ranged from 10 × 10 to 20 × 20. Scanning was performed using a motorized stage. The acquisition time for each point was 6 s. Although 0.5–1.0 s was sufficient to obtain a discernable spectrum, a longer acquisition time was preferred to reduce noise. Throughout the imaging session, the tissue sample remained hydrated with a saline solution (directly covered by saline). The selected areas varied from 1 mm^2^ (100 spectra) to 4 mm^2^ (900 spectra), depending on the size and the shape of the tissue sample. These acquisition times are consistent with state-of-the-art commercial confocal Raman spectroscopy systems^11^.

### Data Analysis and Statistics

For each Raman spectrum, baseline correction was used to remove autofluorescence (msbackadj; MATLAB; Mathworks, Natick, Mass.). Low-SNR spectra were excluded from analysis by examining the intensity ratio between peaks in the CH region (2700–3500 cm^−1^) and peaks in the silent region (1800–2700 cm^−1^), as low-SNR spectra tend to have relatively weaker signals in the CH regions and larger fluctuations in the silent region. A Savitzky-Golay filter with a span of 15 cm^−1^ (smooth; MATLAB; Mathworks, Natick, Mass) was used to denoise the spectra^49^. Next, spectra were normalized according to the intensity around the 1440 cm^−1^ band (CH_2_ bending). Then, by using our pattern recognition algorithm (described below), spectra that were dominated by features of fatty acids were selected for further analysis. After these preprocessing and matching procedures, tissue samples that have more than 10 selected high-quality spectra of fatty acids were determined to be eligible for the following peak analysis. Thus, the number of samples in each group for peak analysis was actually smaller than that in the initial tissue groups, as shown in Tables 1 and 2.

The selection of spectra dominated by fatty acids is crucial in this study as it minimizes the confounding effects from the complex mixture of chemicals present in the breast tissue. Nevertheless, it is important to note that, by only selecting spectra that were dominated by features of fatty acids, the study could risk excluding tissue regions that are not abundant in fatty acids. This has the potential to introduce bias into the analysis of compositional changes in fatty acids if the composition of fatty acids was dependent on the concentration of fatty acids. However, no such correlation between the composition and concentration of fatty acids has been observed in this study or reported in previous studies. In addition, it is not likely that there was any significant data lost from the selection process as a major proportion of the raw data was found to be dominated by fatty acids and have contributed to final statistical analysis (65% of data from cancerous tissue, 70% for normal appearing and 95% for healthy normal in the human study; 4% for solid tumor, 37% for tumor microenvironment, 81% for tumor macroenvironment, 50% for control in the pre-clinical study). The exception of low concentrations of fatty acids in the solid tumors was explained previously in the Results section.

In the peak analysis, Gaussian-peak fitting was used to decompose an overlapping-peak spectrum into its components (see Supplementary Methods). Peak intensity was then calculated by finding the local peak of the fitted Gaussian line. Analysis based on peak areas was also performed to confirm the robustness of the use of peak height in this study (Supplementary Fig. S4). The measurement of DNA concentration was similar to that of the degree of unsaturation of fatty acids except that no pattern recognition process was involved. Since the peak for DNA (787 cm^−1^) is unique in that spectral region and free from obscuring effects from other main components present in breast tissue (Supplementary Fig. S2), the relative DNA concentration was obtained directly from the peak intensity at 787 cm^−1^ of preprocessed spectra. All spectral analyses were performed in MATLAB.

To determine whether tissue samples from healthy and cancerous subjects had significantly different degrees of unsaturation of fatty acid, a Kruskal-Wallis test was used (kruskalwallis; MATLAB), followed by a multiple comparison test (multcompare; MATLAB) with *α *= 0.05. The Kruskal-Wallis test was chosen over one-way ANOVA because the data do not meet the assumptions of a normal distribution.

### Pattern Recognition Algorithm

#### Library Design

The library for the matching algorithm was constructed using reference spectra obtained from pure chemicals (Sigma-Aldrich) that are the known main components of rat mammary and human breast tissue. These main components included unsaturated fatty acid (oleic acid and linoleic acid), cell nuclei (DNA), cell cytoplasm (actin), collagen (collagen type I, III, IV and V), and cholesterol^35^. The basic elements for the construction of this library are shown in Supplementary Figure S2.

#### Matching Algorithm

Once the library of Raman spectra of the basic chemical elements was generated, a matching algorithm was then used to select a signal vector from the library that best corresponded to an observed Raman spectrum. A similarity coefficient was calculated by using Euclidean distance. A K-nearest neighbors search was then applied to classify the spectrum into one specific group. A threshold was chosen for the similarity coefficient so that only spectra composed of mostly one type of basic chemical remained (see Supplementary Methods and Table S1). Saturated fatty acids were excluded in the analysis because we found saturated fatty acids account for very few of the spectroscopic features observed. In addition, Raman spectra from saturated fatty acids and spectra from unsaturated fatty acids are not sufficiently similar to be represented by one group. Matching results for fatty acids in the rat mammary tissue samples are shown in Supplementary Figure S2. The high degree of similarity between the reference fatty acid spectra and the observed tissue spectra, along with the narrow standard deviation of the extracted tissue spectra, demonstrates the efficiency of the matching algorithm.

One modification was made to the matching algorithm for human studies due to the presence of strong resonant signals from β-carotene in human breast tissue. Since β-carotene is strong in the fingerprint vibration region but almost silent in the CH vibration region, and fatty acids have unique spectroscopic features in the CH region compared with other major basic components (Supplementary Fig. S2), fatty acids acquired from human breast tissue were classified based on the CH vibration region.

#### Model Fitting

Previous studies showed that β-carotene is extremely lipophilic and often found in conjunction with fat throughout the human breast^35^. To avoid possible signal interference between β-carotene and fatty acids, multiple linear regression was employed prior to peak analysis to retrieve the relative concentration of fatty acids and β-carotene within a single spectrum (lsqnonlin; MATLAB). The fatty acid information could then be extracted by subtracting the contribution of β-carotene from the total Raman intensity. Model fits to a sample Raman spectra that were classified as fatty acid are shown in Supplementary Figure S2. The flatness of the residual spectra demonstrates that β-carotene and unsaturated fatty acids account for the main chemical components contributing to the measured spectrum.

The basis spectra for the model were acquired from pure β-carotene, oleic acid, and linoleic acid. Because oleic acid and linoleic acid have a very high degree of similarity (Supplementary Fig. S2), they were not sufficiently orthogonal to be easily differentiated in the multiple linear regression. In addition, to avoid overfitting, it is important to involve as few elements as possible while retaining important spectroscopic features. Hence, it was reasonable to use one basis element to represent both monounsaturated fatty acids and PUFAs in the model by extracting their common spectroscopic feature. Therefore, instead of using three full Raman spectra, two sets of several Raman peaks were used to implement model fitting: 1005 cm^−1^ (carotenoids), 1154 cm^−1^ (carotenoids), 1515 cm^−1^ (carotenoids), 1440 cm^−1^ (unsaturated fatty acids), 2303 cm^−1^ (carotenoids), 2661 cm^−1^ (carotenoids), 2897 cm^−1^ (unsaturated fatty acids). More detailed information can be found in Supplementary Methods.

## Additional Information

**How to cite this article**: You, S. *et al*. Raman Spectroscopic Analysis Reveals Abnormal Fatty Acid Composition in Tumor Micro- and Macroenvironments in Human Breast and Rat Mammary Cancer. *Sci. Rep.* **6**, 32922; doi: 10.1038/srep32922 (2016).

## Supplementary Material

Supplementary Information

## Figures and Tables

**Table 1:** Rat mammary tissue samples used in this study.

Rat Study	Type	Rats Planned^*^	Samples planned^*^	Rats used^*^	Samples used^*^
Experiment	Injected with NMU				
	Tumor sites	5	10	1	1
	Surrounding tissue 1 cm away from center of visible tumor	5	17	5	17
	Mammary tissue more than 3 cm away from the center of visible tumor	5	13	5	13
Control	Injected with PBS				
	Healthy mammary tissue	5	37	5	35
Total	77				66

^*^“Rats/Samples planned” refers to tissue samples from which the data have been collected. “Rats/Samples used” refers to the tissue samples that were found to have more than 10 fatty acid-dominated spectra (>10) through the matching algorithm and therefore contributed to the statistical analysis (see Methods).

**Table 2:** Human breast tissue samples used in this study.

Human Study	Type	Patients planned*	Samples planned*	Patients used*	Samples used*
With Breast Cancer	Breast cancer surgery				
	Cancerous tissue from breast cancer patients	7	52	4	22
	Normal-appearing tissue from breast cancer patients	7	29	7	23
Without Breast Cancer	Breast reduction surgery				
	Normal tissue from cancer-free patients	6	38	6	38
Total	119				83

^*^“Patients/Samples planned” refers to tissue samples from which the data have been collected. “Patients/Samples used” refers to the tissue samples that were found to have more than 10 fatty acid-dominated spectra (>10) through the matching algorithm and therefore contributed to the statistical analysis (see Methods).
