# Supplementary material for: Raman Spectroscopic Analysis Reveals Abnormal Fatty Acid Composition in Tumor Micro- and Macroenvironments in Human Breast and Rat Mammary Cancer
Source: Sci Rep. 2016 Sep 6;6:32922. doi: 10.1038/srep32922 (PMC5011773; doi:10.1038/srep32922)
Supplement: Supplementary Information [file srep32922-s1.pdf]

Raman Spectroscopic Analysis Reveals Abnormal Fatty Acid Composition in Tumor Micro- and Macroenvironments in Human Breast and Rat Mammary Cancer

Sixian You[1,2], Haohua Tu[1], Youbo Zhao[1], Yuan Liu[1,2], Eric J. Chaney[1], Marina Marjanovic[1,2], Stephen A. Boppart[1,2,3,4,*]

[1]*Beckman Institute for Advanced Science and Technology, University of Illinois at Urbana-Champaign*

[2]*Department of Bioengineering, University of Illinois at Urbana-Champaign*

[3]*Department of Electrical and Computer Engineering, University of Illinois at Urbana-Champaign*

[4]*Department of Internal Medicine, University of Illinois at Urbana-Champaign*

Supplemental Materials:

Table of contents:

1. Supplementary Figures
2. Supplementary Tables
3. Supplementary Methods
4. References

Supplementary Figure S1

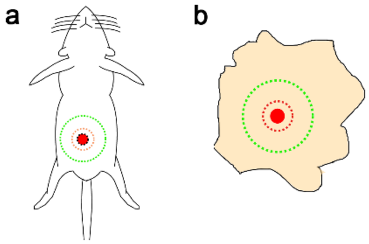

Supplementary Figure S1. Diagram for rat and human tissue selection. (a) A rat model of mammary cancer was used to evaluate the impact of cancer development on spatial locations with varying distance from the primary tumor. The data collected from cancerous rats were divided into three groups. Group 1: tumor sites, which were harvested within solid tumor (red circle with black dashed outline); Group 2: tumor microenvironment, which was surrounding tissues within 1 cm away from the center of the tumor (within the orange dashed circle); Group 3: tumor macroenvironment, which included tissue collected more than 3 cm away from the center of tumor (outside of the green dashed circle). Data for the control group were collected from similar locations in the mammary gland for comparison. **(b)** Human breast tissue was used to validate the findings in rats. Group 1: cancerous tissue, which was identified as tumor (red circle) or non-tumor tissue bordering the tumor (within the red dashed circle) by a certified pathologist. Group 2: normal appearing tissue, which was at least 5 cm away from the primary tumor (outside of the green dashed circle) and labeled as normal by a pathologist. The drawing is for visual representation and is not drawn to scale.

Supplementary Figure S2

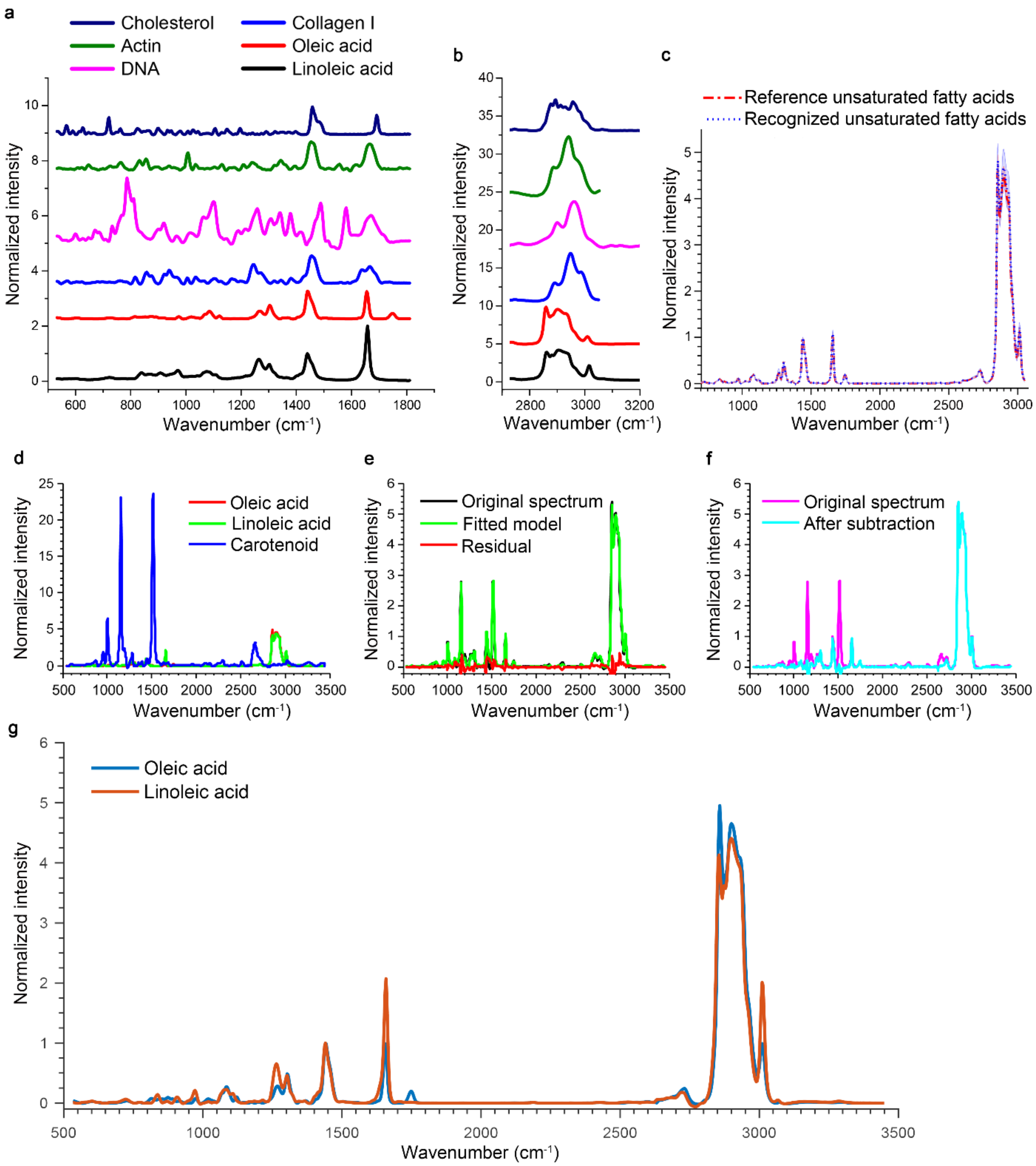

Supplementary Figure S2. Extraction of fatty acid-dominated spectra. (a) Six pure chemicals that are the main contributors to Raman signals from mammary tissue in the fingerprint region and **(b)** CH region. All the spectra were normalized to the intensity at 1440 cm^{-1} and displayed as

stacked lines by Y offset. **(c)** Classification of fatty acids by pattern recognition algorithm. The library of unsaturated fatty acids includes multiple spectra from pure oleic acid and linoleic acid, which most of the unsaturated fatty acids resemble in their Raman spectra. The standard deviation is represented by the shaded area. The small standard deviation demonstrates the high precision of our classification methods for unsaturated fatty acids. **(d-f)** Removal of carotenoids from spectra by model fitting. **(d)** Raman spectra of three pure chemical components involved in the model. **(e)** Results of model fitting. Common peak signatures of the two fatty acids were used as one element and the unique peak signatures of carotenoid were used as the other basic element. **(f)** Based on the model fitting coefficients, the fatty acid content is extracted by subtracting the portion of carotenoids from the raw spectrum. **(g)** Comparison of spectra from oleic acid and linoleic acid.

Supplementary Figure S3

(1) Oleic acid

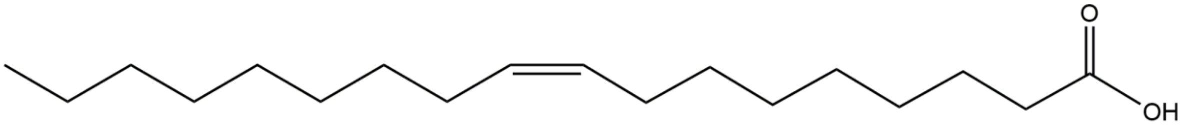

(2) Linoleic acid

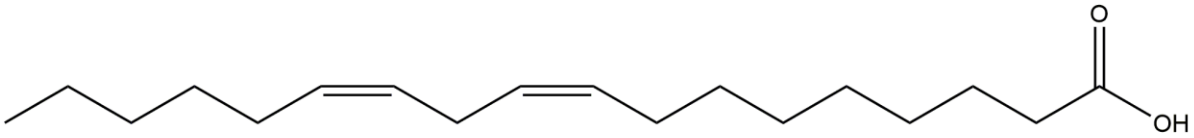

(3) Palmitoleic acid

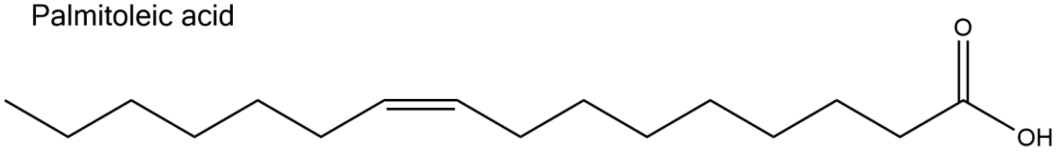

Supplementary Figure S3. Chemical structures of oleic acid, linoleic acid and palmitoleic acid.

Supplementary Figure S4

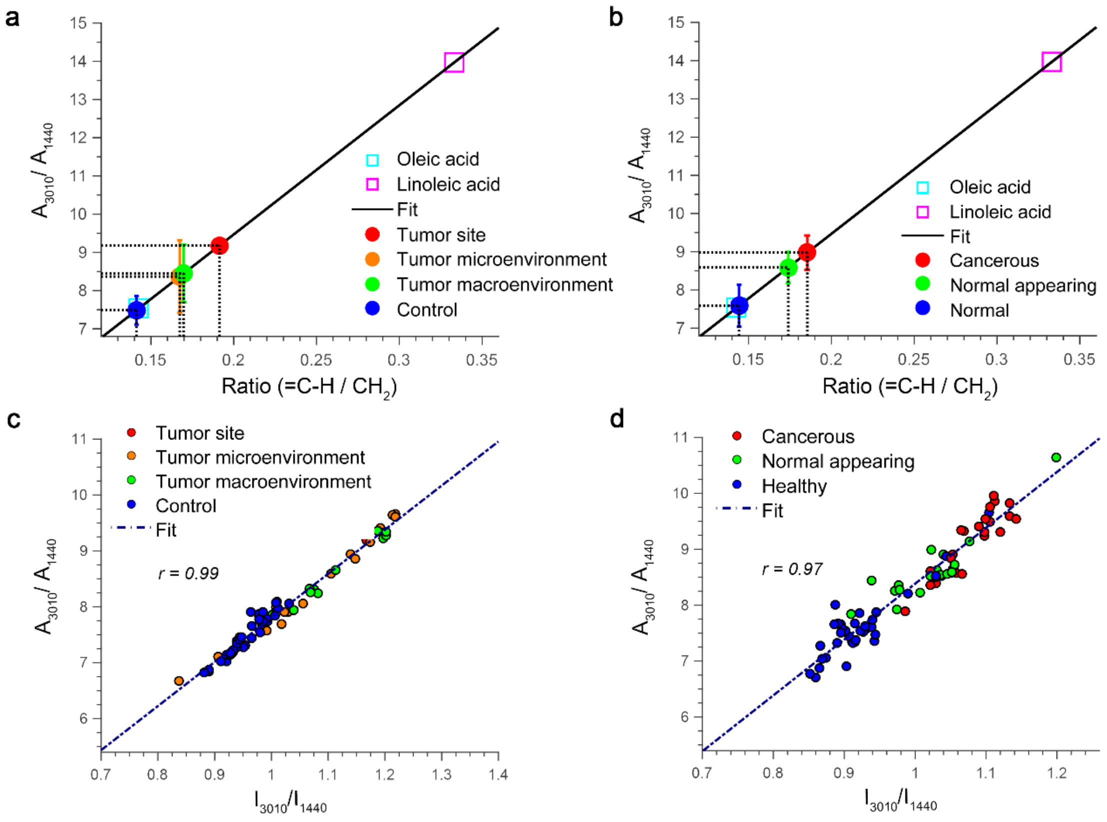

Supplementary Figure S4. Quantitative analysis by peak area and correlation between peak height and peak area. (a) Statistical analysis based on peak area of data obtained from rat and **(b)** human. **(c)** Correlation plot of peak height and peak area for rat and **(d)** human.

Supplementary Table S1. Assignments of the Raman bands used in kNN search.

Wavenumber (cm^{-1})	Assignment[1]
787	DNA<br>Can be taken as a measure for the relative quantity of nucleic acids present
816	C-C stretching of collagen
855	Proline
936	Proline (collagen type I)
1001	Phenylalanie (collagen)
1031	C-N stretching of proteins
1127	C-N stretching of proteins
1243	Collagen
1303	CH_2 deformation of lipids
1312	CH_3CH_2 twisting mode of collagen
1342	DNA/RNA
1581	C=C bending mode of phenylalanine
1655	C=C stretching of lipids
1745	C=O stretching of lipids

Supplementary Methods

Gaussian Peak Fitting

In order to determine the individual vibrational modes that contribute to the Raman signal at 3010 cm^{-1}, the spectra were deconvolved by using a peak-fitting routine (peakfit; MATLAB; Mathworks, Natick, Mass). Although fitting with a large number of peak components usually produces a nearly perfect residual, such models tend to be useless for interpretation and prone to overfitting. Thus, to make the deconvolution scientifically meaningful, the number and the location of major peak components were predicted based on the prior knowledge about the chemical structure and acquired line profile of fatty acids. More specifically, the peak at 2930 cm^{-1} (CH_3 band of lipids) and the peak at 3010 cm^{-1} (C=C band of lipids) were manually selected to fit the spectrum with no restrictions on the peak width. With these settings, peak fitting was then performed to retrieve the contribution from individual bands to the Raman signal. Its performance was evaluated as the percentage of the root mean square of the difference between the sample spectrum and the fitted model with an average value of 2.4%. In addition, to test the robustness of the peak analysis, analyses based on peak areas were implemented and compared to the peak intensity analysis that was employed in this study, as shown in the Supplementary Figure S4. The strong correlation between the value of the peak height and the peak area, as well as the lack of discrepancy between the results of the two methods, shows that the use of peak height is sufficiently robust for this study.

Matching algorithm

The kNN classification was applied to assign a class label to the sample spectrum based on the basis spectra represented by the k (k=3) closest neighbors of the feature space[2]. In order to determine the optimal value of k, 300 spectra were manually labelled and used in a ten-fold cross-validation. Also, as the performance of kNN methods could easily suffer from the curse of dimensionality[3], a total of 14 spectral features were selected based on their uniqueness to each chemical component and their impact on the final classification. The assignments for these Raman bands can be found in the Supplementary Table S1.

After the application of the kNN classifier, to ensure the dominance of fatty acids in each spectrum, every input data x that has a distance larger than a preset distance l (l =0.3) to the nearest training sample in the corresponding class was classified as "mixture" and excluded from the data analysis. It was observed that a threshold too large would include spectra that do not appear dominated by fatty acids while a threshold too small would reject a significant portion of data. Thus, the optimal value of l is reached by maximizing the number of spectra that were dominated by fatty acids without inclusion of any mixture.

References

1. Movasaghi, Z., Rehman, S. & Rehman, I. Raman spectroscopy of biological tissues. *Appl. Spectrosc.* **42,** 493–541 (2007).
2. Cover, T. & Hart, P. Nearest neighbor pattern classification. *Inf. Theory, IEEE Trans.* **13,** 21–27 (1967).
3. Beyer, K., Goldstein, J., Ramakrishnan, R. & Shaft, U. When is 'nearest neighbor' meaningful? *Database Theory—ICDT'99* 217–235 (1999).
